# Vitamin C Inhibits Metastasis of Peritoneal Tumors By Preventing Spheroid Formation in ID8 Murine Epithelial Peritoneal Cancer Model

**DOI:** 10.3389/fphar.2020.00645

**Published:** 2020-05-12

**Authors:** Yayun Xu, Xing Guo, Ganyu Wang, Changkuo Zhou

**Affiliations:** ^1^Department of Hepatobiliary and Pancreatic Surgery, Minhang Hospital, Fudan University, Shanghai, China; ^2^Department of Pharmacy, People’s Hospital of Rizhao, Rizhao, China; ^3^Department of Pediatric Surgery, Qilu Hospital, Shandong University, Jinan, China; ^4^Department of Urology, Qilu Hospital, Shandong University, Jinan, China

**Keywords:** vitamin C, epithelial ovarian cancer, multicellular spheroid, macrophages, metastasis

## Abstract

High mortality is associated with exclusively metastasis within the peritoneal cavity among patients with epithelial ovarian cancer that is the most lethal gynecologic cancer. There is an unmet need to develop more effective therapies to prevent metastasis of peritoneal cancer. Multicellular spheroid formation, during which cancer cells migrate and adhere to tumor-associated macrophages, is a critical step of ovarian cancer metastasis. Here, we showed that vitamin C inhibited spheroid formation and metastasis in ID8 ovarian cancer-bearing mice. We further found that vitamin C treatment decreased the levels of M2 macrophages in tumor nodules and suppressed the epithelial-mesenchymal transition (EMT). *In vitro* studies revealed that vitamin C inhibited proliferation, arrested cell cycle, attenuated migration, and prevented the spheroid formation of ID8 ovarian cancer cells. Vitamin C induced apoptosis of ID8 cells, which was confirmed by membrane potential collapse, cytosolic calcium overload, ATP depletion, and caspase-3 activation in vitamin C-treated cells. Intriguingly, vitamin C treatment caused striking morphological change and apoptosis of macrophages. The presented proof of concept study strategically identifies new anticancer mechanisms of vitamin C.

## Introduction

Epithelial ovarian cancer (EOC), a highly metastatic disease, is the most lethal malignancy of gynecologic origin (Weaver et al., 2018). At the time of diagnosis, most patients with EOC present at an advanced stage (stage III or IV) and have a poor prognosis attributable to widespread intraperitoneal metastases (Wentzensen et al., 2016; Timmermans et al., 2018). The first-line therapy for advanced EOC is cytoreductive surgery and systemic chemotherapy with a platinum analog plus paclitaxel (Vergote et al., 2013; Yu et al., 2015). Despite a high initial response rate, almost all patients ultimately develop platinum/paclitaxel-resistant relapses (Vergote et al., 2013; Mirza et al., 2016); survival rates of patients with EOC have improved only modestly over the past 30 years (Hoskins and Gotlieb, 2017). Thus, the development of effective therapies is essential for treating EOC. Tumor-associated macrophages (TAMs) promote spheroid formation during metastasis of EOC and constitute a central component of spheroid, in which TAMs support tumor cell migration, survive and proliferation by secreting EGF (Yin et al., 2016); therefore, TAMs are attractive targets in EOC treatment (Krishnan et al., 2018).

Vitamin C, a known water-soluble antioxidant, enhances the activity of multiple human enzymes as a cofactor, and contributes to energy metabolism, collagen synthesis, iron absorption, and development of the nervous system in the body (Portugal et al., 2017; Granger and Eck, 2018). Studies over the past decades have consistently demonstrated that vitamin C selectively kills cancer cells and intravenous pharmacological doses of vitamin C improve the efficacy of current anticancer treatment strategies (Yun et al., 2015; Schoenfeld et al., 2017; Gillberg et al., 2018). More recently, studies have shown that vitamin C regulates hematopoietic stem cell function and suppresses the development of leukemia (Agathocleous et al., 2017; Cimmino et al., 2017). Ma and co-workers have recently shown that vitamin C exerts cytotoxic activity against ovarian cancer cells, augments the efficacy of chemotherapies in preclinical ovarian cancer models, and reduces chemotherapy-associated toxicity in ovarian cancer patients (Ma et al., 2014). Although antitumor activities of vitamin C have been extensively studied, the influence of vitamin C on tumor microenvironment remains largely underexplored, and the effects of vitamin C on macrophages and spheroid formation during metastasis of EOC have not been revealed. In this report, using a syngeneic orthotopic and immunocompetent mouse model of epithelial ovarian cancer (ID8) (Su et al., 2010; Duraiswamy et al., 2013), we tested the effects of vitamin C on tumor progression of EOC by intraperitoneal injection and explored the mechanisms of action of vitamin C.

## Materials and Methods

### Materials

Vitamin C (purity: > 99.9%) was purchased from Shandong Xinhua pharmaceutical CO., ltd. DMEM Medium, Fetal Bovine Serum (FBS), Wright-Giemsa staining, and Albumin Bovine V were purchased from MACGENE. RBC lysis Buffer, Sulforhodamine B (SRB), RNase A, Propidium Iodide (PI), and rat IgG were purchased from Solarbio. Triton X-100 solution, Ca^2+^ specific fluorescent probe Fluo-4/AM, JC-1 Staining Kit, One Step TUNEL Apoptosis Assay Kit, and ATP Determination Kit were purchased from Beyotime. FITC Rat Anti-Mouse CD11b was purchased from BD Biosciences. APC Rat Anti-Mouse CD206 was purchased from Miltenyi Biotec. Western blot Antibody Diluent was purchased from Epizyme. Western Bright™ ECL and Western Bright™ Peroxide were purchased from Advansta. Goat Anti-Rabbit IgG (H+L) HRP was purchased from Affinity Biosciences. Vimentin (5G3F10) Mouse mAb, Cleaved Caspase-3 (Asp175) Antibody (Alexa Fluor^®^ 488 Conjugate) and β-actin (13E5) Rabbit mAb were purchased from Cell Signaling Technology. E-cadherin Rabbit PolyAb was purchased from Proteintech.

### Mice

All experiments were performed on 8–10 weeks old female mice. C57BL/6 mice were obtained from laboratory animal center of Shandong University. The Animal Care Committee of Shandong University School of Basic Medical Sciences approved all mouse protocols (NO.ECSBMSSDU2018-2-022).

### Cell Lines and Cell Culture

ID8 cell line (a mouse ovarian epithelial cell line) was kindly provided by Dr. K.F. Roby (Department of Anatomy and Cell Biology, University of Kansas Medical Center, Kansas City, KS) (Roby et al., 2000). ID8 cells were cultured in DMEM medium supplemented with 10% FBS and 1% Insulin-transferrin-selenium (ITS). The L-929 cells were purchased from Shanghai Cell Bank, Chinese Academy of Sciences, and cultured in DMEM medium supplemented with 10% FBS and sodium pyruvate (0.11g/L). All of these cells were maintained at 37°C in a humidified atmosphere of 5% CO_2_.

### *In Vivo* Murine ID8 Ovarian Cancer Model and Vitamin C Treatment

Mice were given an intraperitoneal injection containing 5 × 10^6^ ID8 cells. Two weeks after tumor inoculation, vitamin C (2 g/kg, 4 g/kg) in 400 μl of PBS or PBS was administered intraperitoneally twice daily for 6 weeks. The mice were euthanized and examined for tumor loads by counting the number of tumor nodules on the parietal peritoneal surfaces and diaphragm. Ascitic fluid was collected and the ascitic fluid volume was measured. The number of nucleated cells in ascitic fluid were determined. The nucleated cell counts were expressed as the average number of cells per animal.

### Wright-Giemsa Staining

The tumor spheroids were examined by Wright-Giemsa staining. Ascitic fluid was collected from ID8 tumor-bearing mice. The cells were harvested by centrifugation, and the red blood cells were lysed by RBC lysis buffer. The cells were washed, resuspended in PBS, smeared on slides, and stained with Wright-Giemsa. The tumor spheroids were photographed and counted under a microscope.

### Analysis of Tumor Spheroid Disruption *In Vitro*

After ID8 model mice were sacrificed, ascitic fluid was drawn from abdominal cavity by injector. The cells in ascitic fluid were washed with PBS, centrifuged, resuspended in DMEM with 10% FBS and 10% L-929-conditioned medium, and plated in 6-well culture plates at a density of 0.3ml ascitic fluid per well. The cell culture medium was added different dose of vitamin C, and then the cells were cultured for 48 h. The multicellular spheroids morphology and number were photographed by light microscopy.

### Spheroid Formation *In Vitro*

The mice were injected intraperitoneally with 1 ml sterile starch mixtures to recruit macrophages to peritoneal cavity. At day 3 post-starch mixtures injection, mice were sacrificed and 20 ml DMEM medium was injected intraperitoneally to collect macrophages. Agarose was dissolved in DMEM medium with a concentration of 1.5% and coated the bottom of 24-well flat-bottom plates with 500 μl agarose solution. After solidification, 1×10^3^ macrophages and 1×10^4^ ID8 cells with 1 ml DMEM medium containing 10% L-929 supernatant were mixed, and cultured in plate at 37°C in a humidified atmosphere of 5% CO_2_. The cell culture medium was added different dose of vitamin C, and then the cells were cultured for 48 h. The multicellular spheroids were photographed and counted by light microscopy.

### Western Blot

After mice were sacrificed, ID8 tumor nodules were collected. Tumor nodules protein lysates from different group were collected in RIPA buffer (50 mM Tris-hydrogen chloride pH 7.4, 150 mM NaCl, 1% NP-40, 0.5% sodium deoxycholate, 0.1% SDS) supplemented with complete protease inhibitor cocktail using a tissue homogenizer. Protein was prepared by standard western blot techniques. After blocked with 5% BSA in PBS with 0.5% Tween-20, membranes were incubated with specific primary antibodies (dilution was 1:1,000) at 4°C for 12 h. The membranes were probed with horseradish peroxidase (HRP)-tagged secondary antibodies (dilution was 1:3,000) for 1h at room temperature and then incubated with ECL. Western blot quantification was performed on scanned films using ImageJ software.

### Flow Cytometry Analysis of M2 Macrophages

After ID-8 tumor nodules were collected from different groups, single-cell suspensions of tumor nodes were obtained using digestion solution containing collagenase IV and DNase-I. Cells were blocked with 10 μg/ml rat IgG at 4°C for 10 min and stained with FITC-conjugated anti-CD11b (dilution was 1:100) and APC-conjugated anti-CD206 antibodies (dilution was 1:100) for 30 min at room temperature. Flow cytometry was performed on the Beckman Cytoflex FCM equiped with CytExpert software.

### Sulforhodamine B Assay for Proliferation

Cell proliferation was tested by SRB assay. ID8 cells (3,000 cells) were seeded in 96-well plates in DMEM medium supplemented with 10% FBS and 1% ITS and incubated overnight. The cells were cultured for 72 h with different concentrations of vitamin C. All media were removed and replaced with 100 μl 10% trichloroacetic acid for 1 h. After washing, the fixed cells were stained 100 μl 4% SRB for 30 minutes and then washed with 1% acetic acid. Tris base solution (150 μl, 10 mM) was added to each well and the absorbance was measured at 560 nm using a plate reader.

### Flow Cytometric Detection of Apoptosis Using TUNEL Staining

Terminal deoxynucleotidyl transferase-mediated dUTP nick end labeling (TUNEL) staining was performed to detect the apoptosis of ID8 cells, as described previously with minor modifications (Angelillo-Scherrer et al., 2008). ID8 cells (1 × 10^5^/well) were seeded in a 6-well plate and incubated overnight. Vitamin C was added to the culture, and incubated at 37°C for 24 h. Cells were collected, washed, fixed in 4% paraformaldehyde, permeabilized with 0.1% Triton X-100 solution, incubated with Terminal Transferase and FITC-dUTP. The fluorescence intensity of cells was measured on flow cytometry.

### Measurement of Intracellular Free Ca^2+^

Intracellular free Ca^2+^ levels in ID8 cells were measured using Ca^2+^ specific fluorescent probe Fluo-4/AM as described recently (Jin et al., 2015). ID8 cells were treated with vitamin C for 24 h and loaded with 5 μM Fluo-4/AM for 1 h at room temperature. After incubation, cells were harvested, washed, and analyzed by flow cytometry.

### Mitochondrial Membrane Potential Assay

Mitochondrial membrane potential (ΔΨm) analysis was conducted as described previously (Jin et al., 2015). Briefly, ID8 cells were treated with vitamin C for 24 h. The cells were harvested and incubated with JC-1 at 37°C for 20 min. Stained cells were washed and analyzed by flow cytometry.

### Measurement of Intracellular ATP

ID8 cells were plated into 6-well plates for 24 h prior to the experiment. The cells were treated with different concentrations of vitamin C for 24 h. The cells were lysed by lysis buffer (200 μl/well). Then, cell debris was removed by centrifugation and supernatant fractions were retained for ATP assay. ATP levels of cell lysates were assessed using the ATP Determination Kit (Beyotime) according to the manufacturer’s protocol.

### Measurement of Intracellular Cleaved Caspase-3

Intracellular cleaved caspase-3 was determined by flow cytometry with cleaved Caspase-3 (Asp175) antibody, as described previously (Jin et al., 2015). After treatment with vitamin C for 24 h, the cells were trypsinized, collected by centrifugation, washed twice with PBS, and fixed with 4% paraformaldehyde. The fixed cells were treated with 0.1% Triton X-100, blocked with 1% BSA, and incubated with cleaved caspase-3 (Asp175) antibody (Alexa fluor 488 conjugate, dilution was 1:100) for 30 min. Stained cells were washed with PBS twice and analyzed by flow cytometry.

### Isolation of Bone Marrow-Derived Macrophages (BMDMs) and Vitamin C Treatment

Bone marrow-derived macrophages were harvested and cultured as previously described (Jing et al., 2018). Briefly, mice were euthanized, their femurs were dissected and cut at both ends, and the bone marrow was flushed into DMEM. The RBCs were lysed in Red Blood Cell Lysis Buffer at 4°C. Then, the nucleated cells were washed and resuspended in growth medium consisting of DMEM with 10% FBS and 10% L-929 cell-conditioned medium. The cells were counted, and plated in 6-well culture plates at a density of 1.5 × 10^5^ cells per well. After 3 days, the plates were washed with sterile PBS in order to remove the non-adherent cells. The adherent population was further incubated in macrophage growth media (DMEM + 10% FBS + 10% L-929 cell-conditioned medium) for 4 days. The cells were then treated with vitamin C at different concentrations for 24 h. The cells were photographed by light microscopy, and the rounded cells were counted. The apoptotic cells were determined by TUNEL staining. Intracellular free Ca^2+^ levels in BMDMs were measured using Ca^2+^ specific fluorescent probe Fluo-4/AM. Intracellular ATP levels were assessed using the ATP Determination Kit (Beyotime).

### Cell Cycle Analysis

Cell cycle distribution was assayed according to the method of Li et al. (2014). ID8 cells were treated with vitamin C for 24 h. Cells were harvested by trypsinization, washed with PBS, fixed with ice-cold 70% ethanol in PBS, and kept at -20°C for at least 30 min. After washing twice with PBS, cells were resuspended in 200 μg/ml RNase A at room temperature for 30 min. The resulting cells were incubated with 50 mg/ml PI at 4°C for 30 min. The cells were then subjected to flow cytometry, and DNA histograms were analyzed using CytExpert software.

### *In Vitro* Migration Assay

Cell migration was assessed by the wound-healing scratch assay. Briefly, ID8 cells (5 × 10^4^) were seeded in 24-well plates. After the cells reached confluence, an artificial wound was created by manually scraping the confluent monolayer cells with a sterile 200 μl pipette tip. After washing, the cells were incubated in the presence or absence of vitamin C, and the status of the gap closure was observed and photographed.

### Ethics Statement

The animal study was reviewed and approved by the Animal Ethical Committee of Basic Medical Sciences, Shandong University.

### Statistical Analysis

Analysis of variance (ANOVA) was performed using Prism software (GraphPad Software, Inc.). P values < 0.05 were considered statistically significant, and P < 0.01 was regarded as highly significant.

## Results

### Vitamin C Suppresses Intraperitoneal Metastasis in Mice Bearing ID8 Ovarian Cancer

**Figure 1:**
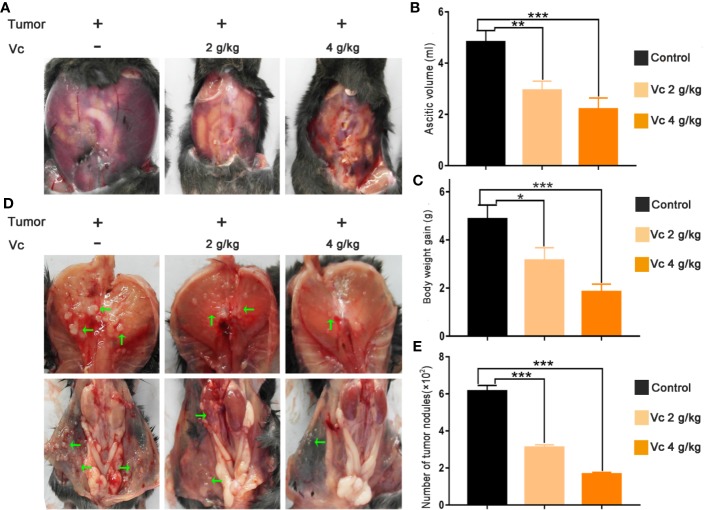
Vitamin C reduces intraperitoneal metastasis and malignant ascites in mice bearing ID8 ovarian cancer. **(A)** Representative images of bloody ascites derived in peritoneal cavity from control and vitamin C treatment groups. **(B)** Ascites volume in different groups. **(C)** Body weight gain in different groups. **(D)** Representative images of tumor nodules in diaphragm and peritoneal wall. **(E)** Metastatic dissemination in diaphragm and peritoneal wall was assessed by counting metastatic colonies in individual mice. Data are expressed as the mean ± SEM; n=8 mice per group for one out of three independent experiment. *p < 0.05, **p < 0.01, ***p < 0.001.

Peritoneal injection of serous ovarian cancer ID8 cells is an established model for the study of metastases, malignant ascites, and cancer-associated spheroid; this model mimics stage III/IV ovarian carcinoma and is ideally suited to study the efficacy of ovarian cancer therapies (Duraiswamy et al., 2013; Yin et al., 2016; Wieland et al., 2017). Gross metastatic intraperitoneal nodules arise about 4 weeks after injection of ID8 cells, and tumor and ascites promptly accumulate, leading to weight gain of mice (Duraiswamy et al., 2013). We initially tested whether vitamin C affects metastases of ovarian cancer upon treatment *in vivo* of ID8 tumor-bearing mice. We treated 14-day established peritoneal ID8 tumors by intraperitoneal vitamin C (2 g/kg, 4 g/kg) injection twice daily for 6 weeks and analyzed the residual peritoneal tumor deposits. We found that ID8 tumor-bearing mice produced a large of amount of ascitic fluid and had substantial tumor growth in the peritoneal cavities (**Figure 1**). There was a significant decrease of malignant ascites and a body weight reduction in mice treated with vitamin C (**Figures 1A–C**). In accordance with observations of ascites and body weight, vitamin C-treated mice showed a significant reduction in number of tumor nodules on the peritoneal wall and diaphragm compared with control (**Figures 1D, E**). These results suggest that vitamin C possesses superior *in vivo* antitumor properties in a dose-dependent manner in metastasis model of ID8 murine ovarian cancer.

### Vitamin C Prevents Peritoneal Spheroid Formation in ID8 Murine Epithelial Ovarian Cancer Model

**Figure 2:**
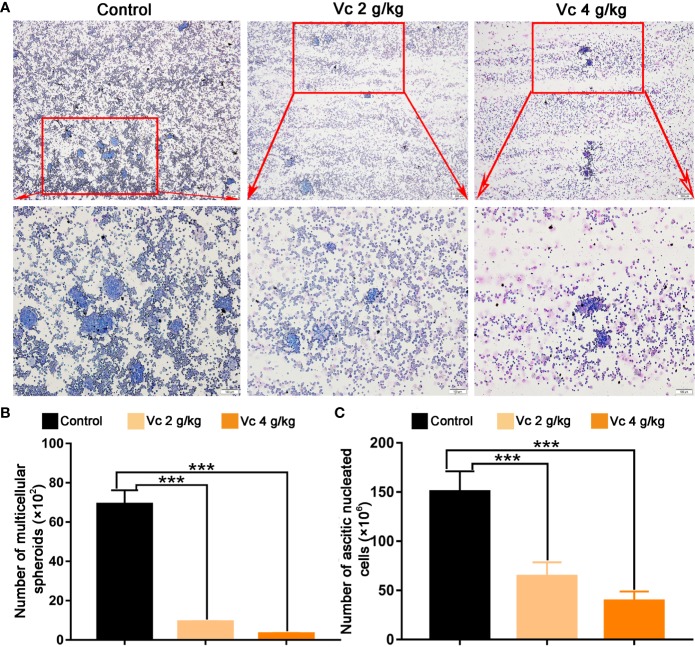
Vitamin C prevents multicellular spheroid formation in ID8 tumor-bearing mice. **(A)** Representative images of multicellular spheroids from the ascitic fluid. upper panels ×40 and lower panels ×100. **(B)** The quantitation of multicellular spheroids from ascitic fluid. Data are mean ± SEM; n=5. ***p < 0.001. **(C)** Ascitic fluid nucleated cell counts in different groups. Data are mean ± SEM; n=8. ***p < 0.001.

**Figure 3:**
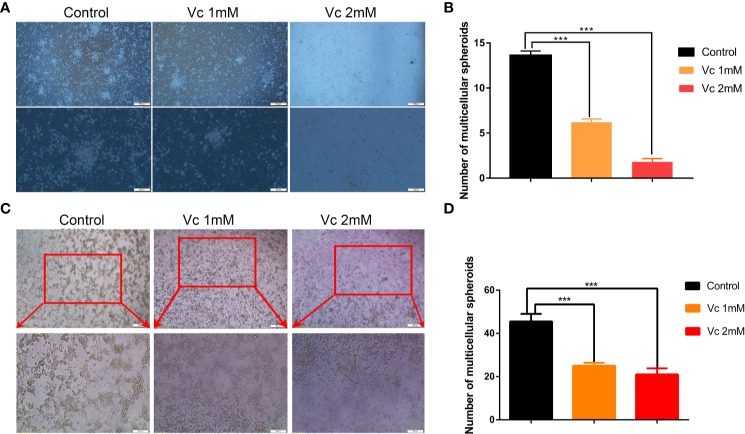
Vitamin C disrupts ascites-derived multicellular spheroid and prevents multicellular spheroid formation *in vitro*. **(A)** Representative images of ascites-derived multicellular spheroids in 6-well plate. upper panels ×40 and lower panels ×100. **(B)** The quantitation of ascites-derived multicellular spheroids in 6-well plate. Data are mean ± SEM; n=5. ***p< 0.001. **(C)** Representative images of multicellular spheroids constructed *in vitro* by mixed ID8 cells and macrophages. upper panels ×40 and lower panels ×100. **(D)** The quantitation of multicellular spheroids constructed *in vitro* by mixed ID8 cells and macrophages. Data are mean ± SEM; n=5. ***p.< 0.001.

**Figure 4:**
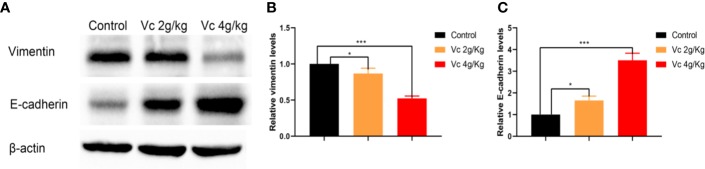
Vitamin C inhibits epithelial-mesenchymal transition (EMT) of ovarian cancer cells in ID8 tumor nodules. **(A)** Western blot analysis of vimentin and E-cadherin in ID8 tumor nodules from ID8 tumor-bearing mice and ID8 tumor-bearing mice treated with vitamin C. **(B, C)** The quantitation of vimentin and E-cadherin levels in different ID8 tumor nodules by using ImageJ software. Data are mean ± SEM; n = 3. *p < 0.05, ***p < 0.001.

**Figure 5:**
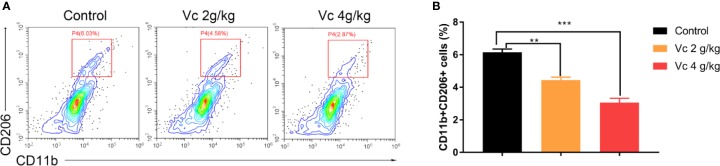
Vitamin C reduces M2 macrophage in ID8 tumor nodules. Flow cytometry analyses of CD11b+CD206+ macrophages from ID8 tumor nodules. **(A)** Representative examples of CD11b and CD206 profiles in ID8 tumor nodules from ID8 tumor-bearing mice and ID8 tumor-bearing mice treated with vitamin C. **(B)** The quantitation of CD11b and CD206 positive macrophages (CD11b+CD206+) from ID8 tumor nodules. Data are mean ± SEM; n = 3. **p < 0.01, ***p < 0.001.

Given that multicellular spheroid formation is an essential step in the peritoneal implantation metastasis for ovarian cancer, we test the effect of vitamin C on multicellular spheroid formation *in vivo* by Wright-Giemsa staining. Interestingly, the number of spheroids significantly decreased in a dose-dependent fashion after vitamin C (2 g/kg, 4 g/kg) twice daily intraperitoneal injection for six consecutive weeks (**Figures 2A, B**). Consistent with the spheroid observation, we found that ascitic fluid nucleated cell counts were lower in vitamin C-treated mice than in control ID8 tumor-bearing mice after vitamin C treatment for six weeks (**Figure 2C**). We then collected the multicellular spheroids from the ascitic fluid and cultured them with vitamin C in 6-well plates. The results showed that vitamin C disrupted the spheroid structure (**Figures 3A, B**). A co-culture of macrophages and ID8 cells was adapted from a previous study (Hirst et al., 2018), using the liquid overlay method on agarose-coated 24-well plates. We found that vitamin C inhibited the spheroid formation *in vitro* (**Figures 3C, D**). Epithelial-mesenchymal transition (EMT) is a process in embryonic development (Aiello and Kang, 2019); tumor cells can reactivate EMT, which enhances cellular mobility and promotes cancer metastasis. A hallmark of EMT is the functional loss of E-cadherin that interferes with spheroid formation in ovarian carcinoma (Davidson et al., 2012). Upregulation of the vimentin is also observed frequently. We found that vitamin C decreased vimentin expression and increased E-cadherin expression in ID8 tumor nodules (**Figure 4**), which indicated that vitamin C suppresses EMT in ovarian cancer cells *in vivo*. M2 tumor-associated macrophages promote EMT (Zhang et al., 2019) and spheroid formation (Yin et al., 2016). Thus, we evaluated the effect of vitamin C on M2 macrophages in ID8 tumor nodules. We observed that vitamin C reduced the M2 macrophages in ovarian tumor microenvironment (**Figure 5**). Our data suggest that vitamin C reduces multicellular spheroid formation in ID8 tumor-bearing mice, and targeting spheroids with vitamin C reveals a strategy for ovarian cancer therapy.

### Vitamin C Inhibits the Proliferation and Induces Cell Cycle Arrest of ID8 Ovarian Cancer Cells

**Figure 6:**
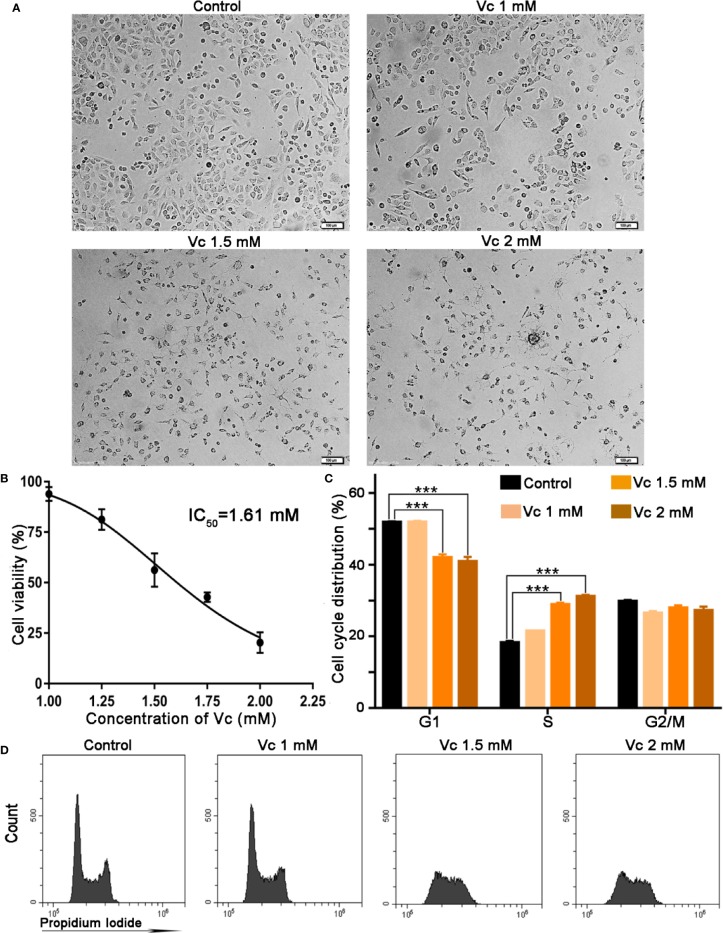
Vitamin C inhibits proliferation and induces cell cycle arrest at S stage in ID8 cells. **(A)** Representative photographs of ID8 cells in different groups. ×100. **(B)** Cytotoxicity of pharmacologic vitamin C on ID8 ovarian cancer cells. Data are mean ± SEM; n=3. **(C)** The statistical data of cell cycle distribution. Data are mean ± SEM; n=4. ***p < 0.001. **(D)** A fluorescence pattern of Propidium Iodide (PI) stained cells.

The multicellular spheroid of ovarian cancer mainly consists of tumor cells and tumor-associated macrophages (Yin et al., 2016; Long et al., 2018). We then examined the biological effects of vitamin C on ID8 cell proliferation by SRB staining. The cells were photographed using phase-contrast microscopy; vitamin C treatment induced distinctive morphological changes in ID8 cells. ID8 cells grew in aggregates with individual cells presenting polygonal and retracted shapes. Cells treated with 1 mM vitamin C for 24 h also grew in groups, but developed unusual shapes. Treatment with 1.5 and 2 mM vitamin C induced striking morphological changes in ID8 cells; the vitamin C-treated cells grew as small and single cells, and displayed prominent long filamentous processes (**Figure 6A**). As illustrated in **Figure 6B**, vitamin C treatment caused a concentration-dependent reduction in proliferation rate of ID8 cells with an IC50 of 1.61 mM; at 2 mM, vitamin C strongly inhibited the proliferation of ID8 cells compared with the control cells. Next, we investigated whether induction of cell cycle arrest could contribute to growth-inhibitory function of vitamin C in ID8 cells. The effects of vitamin C on cell cycle distribution were evaluated by PI staining. The flow cytometric analysis for the DNA content in ID8 cells showed that the cell population in the S phase increased after the cells were subjected to vitamin C (1.5 and 2 mM). Vitamin C (1.5 mM) treatment caused 29.9% cells in S phase as compared with control showing 18.7%. Conversely, G1 phase cell population was decreased to 39.8% as compared with control having 52.1% (**Figures 6C, D**). These experiments suggest that cell cycle arrest contributes to vitamin C-induced growth inhibition of ID8 ovarian cancer cells and confirm the therapeutic effect of vitamin C on ovarian cancer.

### Vitamin C Induces Apoptosis in ID8 Ovarian Cancer Cells

**Figure 7:**
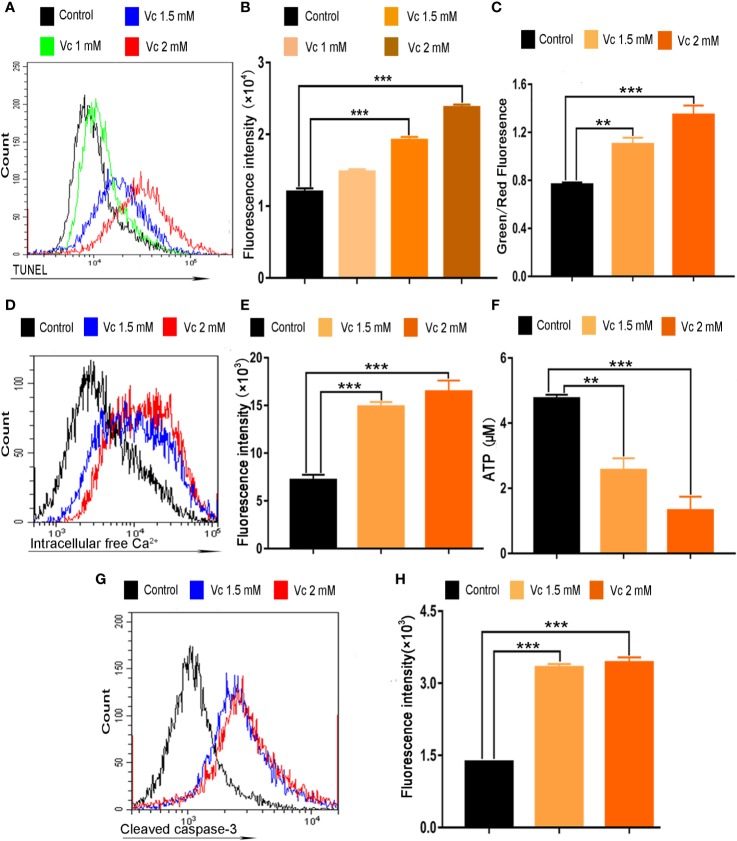
Vitamin C induces apoptosis of ID8 cells. **(A)** The fluorescence pattern of terminal deoxynucleotidyl transferase-mediated dUTP nick end labeling (TUNEL)–stained ID8 cells. **(B)** Cell associated mean fluorescence intensities of TUNEL-stained ID8 cells. **(C)** Effect of vitamin C on mitochondrial membrane potential in ID8 cells. **(D)** The fluorescence pattern of Fluo-4/AM-stained ID8 cells. **(E)** Cell associated mean fluorescence intensities of Fluo-4/AM-stained ID8 cells. **(F)** The levels of ATP in ID8 cells. **(G, H)** ID8 cells were stained with cleaved caspase-3 (Asp175) antibody (Alexa fluor 488 conjugate). **(G)** The fluorescence pattern of ID8 cells. **(H)** Cell associated mean fluorescence intensities. Data are mean ± SEM; n=4. **p < 0.01, ***p < 0.001.

We further explored whether apoptosis induction contributes to cytotoxic activity of vitamin C in ID8 cells. Vitamin C treatment shows a dose-dependent accumulation of cells in apoptosis as measured by TUNEL staining (**Figures 7A, B**). We next focused on the potential mechanisms responsible for vitamin C-induced apoptosis in ID8 ovarian cancer cells. The impairment of mitochondrial integrity and the subsequent loss of mitochondrial membrane potential (ΔΨm) are the early events in the initiation and activation of apoptotic cascades (Jin et al., 2015). To determine whether vitamin C induce mitochondrial disruption in ID8 cells, we carried out a flow cytometry analysis using JC-1 staining and evaluated the fluorescence emission shift from red to green. Mitochondrial depolarization occurring in vitamin C (1.5 and 2 mM)-treated cells is indicated by increase in the green/red fluorescence intensity ratio (**Figure 7C**). Mitochondria uptake Ca^2+^, which has proved to be essential for control the Ca^2+^ concentration in the cytoplasm; ΔΨm provides the driving force for Ca^2+^ accumulation in mitochondria (Jin et al., 2015; Boeckel and Ehrlich, 2018). Therefore, we investigated the role of calcium signaling in vitamin C-induced apoptosis of ID8 cells; cytosolic Ca^2+^ was measured with a calcium indicator dye, Fluo-4/AM. In line with our observation of ΔΨm collapse in vitamin C-treated cells, we found that treatment with vitamin C (1.5 and 2 mM) resulted in an elevation of Ca^2+^ in the cytoplasm of ID8 cells (**Figures 7D, E**). Calcium overload and mitochondrial dysfunction lead to depletion of ATP that is mainly produced in mitochondria (Li et al., 2018; Zhang et al., 2018). We then determined the effect of vitamin C on the content of ATP, and we found that treatment of ID8 cells with vitamin C decreased the levels of ATP (**Figure 7F**). To determine whether vitamin C induces apoptosis of ID8 cells through a mitochondria-dependent caspase pathway, we examined the activation of caspase-3 by flow cytometry using specific antibodies that recognize the cleaved and activated form. As expected, leaved and activated form of caspase-3 was increased in ID8 cells expose to vitamin C (1.5 and 2 mM) (**Figures 7G, H**). Taken together, these data demonstrate that vitamin C displays remarkable anticancer activities in ID8 murine ovarian cancer cells by inducing mitochondrial-dependent apoptosis.

### Vitamin C Causes Apoptosis in Macrophages

**Figure 8:**
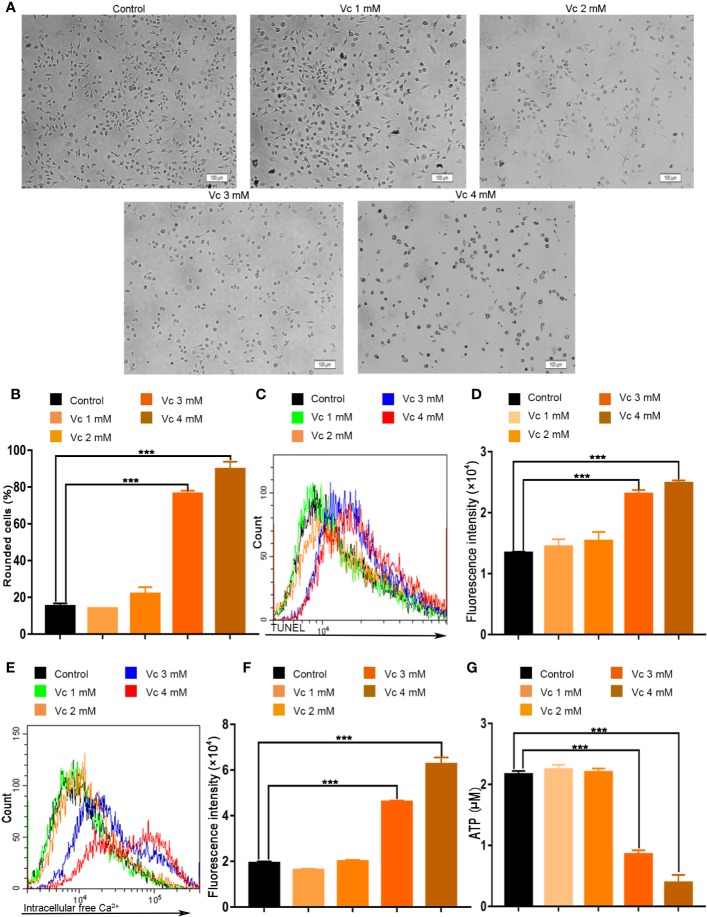
Vitamin C causes apoptosis in macrophages. **(A**, **B)** Primary cultures of macrophages derived from bone marrow of mice. The bone marrow-derived macrophages (BMDMs) were treated with the different dose of vitamin C for 24 h. **(A)** The representative fields were photographed at 100× magnification. **(B)** For quantitative analysis, the percentage of rounded cells was determined. **(C)** The fluorescence pattern of terminal deoxynucleotidyl transferase-mediated dUTP nick end labeling (TUNEL)–stained BMDMs. **(D)** Cell associated mean fluorescence intensities of TUNEL-stained BMDMs. **(E)** The fluorescence pattern of Fluo-4/AM-stained BMDMs. **(F)** Cell associated mean fluorescence intensities of Fluo-4/AM-stained BMDMs. **(G)** The levels of ATP in BMDMs. Data are mean ± SEM; n=3. ***p < 0.001.

Tumor tissue is composed of diverse cell types, including cancer cells and the stromal cells. A considerable number of innate immune cells reside in the tumor microenvironment; TAMs are a major cellular component of the cancer microenvironment and play crucial roles in the regulation of tumor progression in a variety of tumors (Henze and Mazzone, 2016). Macrophages is a component of multicellular spheroids in ovarian cancer (Yin et al., 2016; Long et al., 2018). We hypothesized that the inhibition of multicellular spheroids formation could be the result of impaired macrophages in ID8 tumor-bearing mice after vitamin C treatment. We evaluated the cytotoxic effect of vitamin C on BMDMs from the mice. The status of BMDMs was first observed under the microscopy; vitamin C (3 and 4 mM) treatment of BMDMs from mice was associated with rounded shape (**Figure 8A**). The percentage of rounded cells in vitamin C-treated BMDMs was increased in a dose-dependent manner (**Figure 8B**). In order to further confirm the inhibitory effect of vitamin C on BMDMs, we examined the apoptosis of BMDMs after vitamin C (3 and 4 mM) treatment. We found that vitamin C was able to induce apoptosis in macrophages, as revealed by enhanced TUNEL staining, increased cytosolic calcium, and ATP depletion (**Figures 8C–G**).

### Vitamin C Inhibits the Migration of ID8 Ovarian Cancer Cells

**Figure 9:**
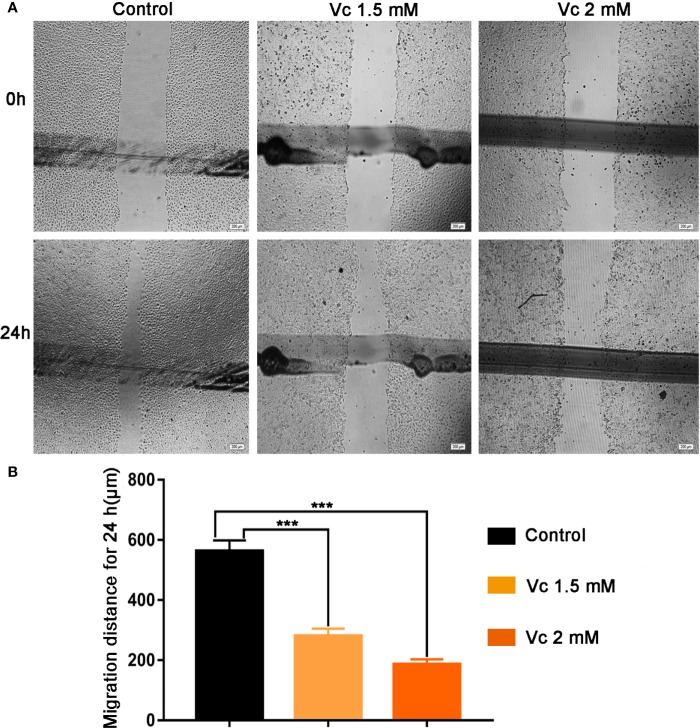
Effect of Vitamin C on ID8 cell migration. Migration of ID8 cells was evaluated using the *in vitro* wound-healing scratch assay. **(A)** Representative photomicrographs of ID8 cells. **(B)** For quantitative analysis, migration distance of cells was determined. Data are mean ± SEM; n=4. ***p < 0.001.

Tumor cell migration is pivotal step for cancer cell dissemination and metastasis, and is controlled by signal-mediated cytoskeletal and cell matrix adhesion remodeling (van Roosmalen et al., 2015; Paul et al., 2017). Ovarian cancer cell migration is an important process in multicellular spheroids formation, during which macrophage-secreted EGF promotes ovarian cancer cell migration toward TAMs and adhesion to TAMs (Yin et al., 2016). To comprehensively evaluate the anticancer activity of vitamin C, we examined the effects of vitamin C on the migration of ID8 ovarian cancer cells. As shown in **Figure 9**, treatment with vitamin C decreased the migratory capability of the ID8 cells as assessed by wound-healing scratch assay.

## Discussion

The high mortality associated with EOC is mainly due to extensive intraperitoneal metastases that are generally unable to be removed completely by surgery (Miranda et al., 2016). Large intraperitoneal tumor burden causes refractory ascites that presents a considerable clinical challenge to the management of ovarian cancer (Kipps et al., 2013). Metastatic dissemination of ovarian cancer involves several steps and occurs in distinct microenvironments that play important regulatory roles in tumor cell behavior, including attachment, survival, migration, invasion, and proliferation. During peritoneal transcoelomic metastasis, ovarian cancer cells detach from primary tumor site, survive in a nonadherent state in the peritoneal fluid, are transported throughout the peritoneal cavity as single cells or multicellular spheroids and attach on the surface of intra-abdominal organs, including the omentum, peritoneum, diaphragm, and small bowel mesentery. Ultimately, metastasis nodules develop on the surface of the peritoneal cavity organs and spread throughout the intraperitoneal region. While in peritoneal fluid, the ovarian cancer cells must survive the hostile environment and resist anoikis that is a specialized form of cell death caused by loss of contact with the extracellular matrix. Cells which do not assemble to form spheroids in suspension are more prone to anoikis; multicellular spheroids protect tumor cells from anoikis and promote proliferation of ovarian cancer cells, which allows for ovarian cancer cells survival during the process of metastasis and promotes ovarian cancer metastasis (Bilandzic and Stenvers, 2014; Yin et al., 2016; Long et al., 2018). Macrophages have long been considered immune cells and play critical roles in tissue inflammation and protecting the organism from infection; they also show great functions in development, homeostasis, and tissue repair (Ginhoux and Guilliams, 2016; Jing et al., 2018). However, TAMs contribute to the pathophysiology of cancer, and high numbers of TAMs in human tumors usually correlate with poor prognosis. Many studies have shown that TAMs can promote tumor progression by stimulating angiogenesis, enhancing cancer cell invasion, suppressing adaptive antitumor immunity, and limiting the efficacy of various forms of anticancer therapies (Henze and Mazzone, 2016; Lewis et al., 2016; Zhu et al., 2017). TAMs promote ovarian cancer cell proliferation and migration, spheroids formation, and tumor growth by secreting EGF at early stage of transcoelomic metastasis of ovarian cancer (Yin et al., 2016). Therefore, macrophages are attractive targets for therapeutic development in ovarian cancer. In solution, vitamin C is readily oxidized to dehydroascorbate (DHA) that is transported intracellularly by GLUT1, causes oxidative stress, and is selectively toxic to cancer cells, particularly to those overexpressing GLUT1 (Yin et al., 2016). Erythrocytes express very high levels of GLUT1, obtain DHA, and reduce it to vitamin C, which keeps DHA levels low and protects cancer cells from vitamin C-induced oxidative stress (van der Reest and Gottlieb, 2016; Zhang et al., 2016). Importantly, intravenous injection of high-dose vitamin C may induce hemolysis in glucose-6-phosphate dehydrogenase-deficient patient (Huang et al., 2014; Quinn et al., 2017). Another risk is that, intravenous administration of high-dose vitamin C exposes the red blood cells to extremely high concentrations of vitamin C, which may promote thrombosis through procoagulant activation of erythrocytes (Kim et al., 2015). In recent years, ideal treatment for EOC has evolved greatly. Clinical studies suggest that intraperitoneal delivery of chemotherapy improves outcomes compared with intravenous administration of chemotherapy in patients with ovarian cancer due to the localized nature of the disease (Miller et al., 2017; van Driel et al., 2018). In our experiments, vitamin C was administered by intraperitoneal injection in ID8 ovarian tumor-bearing mice. The results show that vitamin C inhibits ovarian cancer metastasis *in vivo* and our data indicate that vitamin C suppresses multicellular spheroid formation in ovarian cancer by decreasing M2 macrophages in tumor microenvironment and inducing macrophage apoptosis. Our results reveal a mechanism that helps to explain the anticancer effects of vitamin C in various types cancer.

And then we found that vitamin C inhibits proliferation, arrests cell cycle, and induces apoptosis in ID8. Mitochondria are essential for energy production and calcium homeostasis, and play a key role in modulating apoptosis (Bouchier-Hayes et al., 2005; Shen et al., 2018). Calcium (Ca^2+^) is a ubiquitous and versatile intracellular second messenger; cellular Ca^2+^ homeostasis plays a pivotal regulatory role in many facets of cellular physiology, including transcription, metabolism, contraction, proliferation, differentiation, secretion, and apoptosis. The concentration of cytosolic calcium is tightly regulated, and calcium overload can induce apoptosis (Jin et al., 2015; Boeckel and Ehrlich, 2018). Caspases, a family of cysteine proteases, play a central role in the execution of apoptotic cell death; activation of caspase-dependent cascade is the critical process in apoptosis. Cytochrome c is released from the mitochondrial intermembrane space into the cytoplasm after ΔΨm collapse, which triggers the formation of apoptosome and consequently activates caspase-3, one of the main executors of apoptosis (Lavrik et al., 2005; Cheriyath et al., 2007). Our data indicate that vitamin C induces apoptosis of ID8 ovarian cancer cells through the mitochondrial pathway, including ΔΨm collapse, Ca^2+^ overload, ATP depletion, and caspase-3 activation.

**Figure 10:**
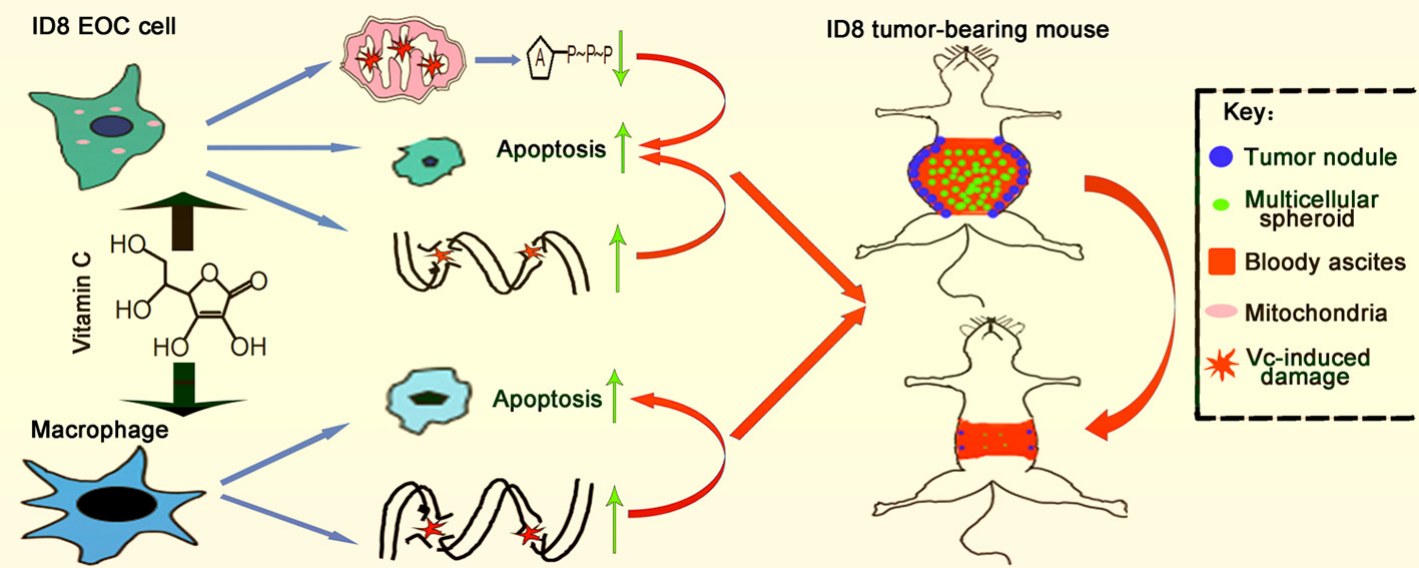
Schematic illustration of vitamin C inhibits metastasis of peritoneal tumors in ID8 murine epithelial peritoneal cancer model.

Collectively, our studies utilize ID8 murine ovarian cancer line, ID8 tumor-bearing mice, as well as BMDMs model, to show the antitumor activity of vitamin C. Vitamin C decreases the levels of M2 macrophages in tumor nodules, suppresses the EMT, prevents multicellular spheroid formation, and suppresses ID8 murine ovarian cancer metastasis in animal tumor model studies. Treatment of ID8 cells with vitamin C inhibits growth, causes cell cycle arrest, induces apoptosis, and decreases the migratory capability in ovarian cancer cells. Importantly, vitamin C induces apoptosis in macrophages (**Figure 10**). Our studies improve our understanding of anticancer effects of vitamin C and provide the rationale for the development of vitamin C-based therapies in ovarian cancer.

## Data Availability Statement

All datasets generated for this study are included in the article/supplementary material.

## Ethics Statement

The animal study was reviewed and approved by The Animal Care Committee of Shandong University School of Basic Medical Science.

## Author Contributions

CZ designed the study and wrote the article. YX and XG performed the animal and cell experiments and wrote the article. GW performed the animal studies and collected data.

## Funding

This work was supported by science and technology development plan of Shandong Province (Grant No. 2014WS0144).

## Conflict of Interest

The authors declare that the research was conducted in the absence of any commercial or financial relationships that could be construed as a potential conflict of interest.

## Abbreviations

BMDMs, Bone marrow-derived macrophages; DHA, Dehydroascorbate; EMT, Epithelial-mesenchymal transition; EOC, Epithelial ovarian cancer; FBS, Fetal Bovine Serum; ITS, Insulin-transferrin-selenium; PI, Propidium Iodide; SRB, Sulforhodamine B; TAMs, Tumor-associated macrophages; TUNEL, Terminal deoxynucleotidyl transferase-mediated dUTP nick end labeling; ΔΨm, Mitochondrial membrane potential.
